# Draft genome sequence of *Vibrio pectenicida* strain FHCF-3, a causative agent of sea star wasting disease in the sunflower sea star (*Pycnopodia helianthoides*), reveals the genetic potential to produce aerolysin-like toxins

**DOI:** 10.1128/mra.00287-25

**Published:** 2025-08-14

**Authors:** Kevin Xu Zhong, Amy M. Chan, Melanie B. Prentice, Yasmine Gouin, Drew Harvell, Alyssa-Lois M. Gehman, Curtis A. Suttle

**Affiliations:** 1 Department of Earth, Ocean and Atmospheric Sciences, The University of British Columbia 175064, Vancouver, British Columbia, Canada; 2 The Hakai Institute, Campbell River, British Columbia, Canada; 3 Friday Harbor Laboratories, University of Washington 7284, Friday Harbor, Washington, USA; 4 Department of Ecology and Evolutionary Biology, Cornell University Department of Ecology and Evolutionary Biology 171526 , Ithaca, New York, USA; 5 Institute for the Oceans and Fisheries, The University of British Columbia, Vancouver, British Columbia, Canada; 6 Department of Botany, The University of British Columbia 98685, Vancouver, British Columbia, Canada; 7 Department of Microbiology & Immunology, The University of British Columbia 8166, Vancouver, British Columbia, Canada; California State University San Marcos, San Marcos, California, USA

**Keywords:** sea star wasting disease, genomes, whole-genome sequencing (WGS), *Vibrio pectenicida*, aerolysin, sunflower sea star (*Pycnopodia helianthoides)*

## Abstract

Here, we report the genomic sequence of *Vibrio pectenicida* strain FHCF-3, isolated from the coelomic fluid of a sunflower sea star (*Pycnopodia helianthoides*) showing signs of wasting disease. The draft genome is 4,368,354 bp with 3,903 coding sequences, including 3 with significant sequence similarity to aerolysin-like toxins.

## ANNOUNCEMENT

Sunflower sea stars (*Pycnopodia helianthoides*) along the Pacific coast have been devastated by sea star wasting disease (SSWD) (1). Here, we report the draft genome of *Vibrio pectenicida* strain FHCF-3, isolated from the coelomic fluid of a sunflower sea star exhibiting signs of SSWD, collected near Friday Harbor, Washington, USA, in February 2024.

Coelomic fluid samples were withdrawn from the sea star coelomic cavity using a syringe fitted with a 26G needle. Samples were transported on ice to The University of British Columbia and spread onto MLB (2, 3) plates (0.05% each of casamino acids, peptone, and yeast extract, 0.3% glycerol, 1.2% agar, in 24 practical salinity units [PSU] seawater) within 8 hours of collection. After incubation at room temperature (ca. 21°C) for 5–7 days, individual colonies were picked and re-streaked repeatedly to achieve axenic clonal cultures of the bacterium. We used whole-genome sequencing (WGS) and phylogenetic analysis to identify FHCF-3 as a new strain of *Vibrio pectenicida*.

To prepare samples for WGS, marine broth (0.5% peptone, 0.1% yeast extract, and 24 PSU seawater) was seeded with clonal colonies and incubated at 18°C for 3 days. Cells were harvested by centrifugation at 11,000 × *g* for 5 minutes and sent to Seqcenter (Pittsburgh, PA) for genomic DNA extraction and hybrid assembly sequencing (Nanopore/Illumina Combo), following Materials and Methods as described previously (4). Briefly, Illumina libraries were prepared with the Illumina DNA Library Prep Kit (Illumina Inc., San Diego, CA) and sequenced on a NextSeq2000 (151 bp paired end), yielding 22,547,344 short reads (Table 1). Nanopore libraries were prepared with the Oxford Nanopore Technologies (ONT, UK) Ligation-Sequencing Kit (no size selection for genomic DNA), sequenced on a MinION (R9.4.1 flow cell), and base called using Guppy v4.2.2, yielding 515,036 long reads (N_50_ = 6,342 bp). Adapters and low-quality reads were trimmed using bcl2fastq v2.19.0 (5) and Porechop v0.2.4 (6) for Illumina and ONT sequences, respectively. Unicycler v0.5.0 (7) was used to perform hybrid assembly of both read types. The draft genome of FHCF-3 is 4,368,354 bp in size, consisting of five contigs (N_50_ = 1,415,317 bp), with an average GC content of 41.5% (Table 1). Analysis using CheckM v1.0.18 (8) revealed that the FHCF-3 genome was 100% complete and had 0.45% contamination compared to other reference genomes in the database.

**TABLE 1:** Summary of the draft genome sequences of *Vibrio pectenicida* strain FHCF-3

Variable	Data
Species	*Vibrio pectenicida*
Strain	FHCF-3
NCBI accession no.	JBLZMR000000000
Isolation source	Sunflower sea star (*Pycnopodia helianthoides*) coelomic fluid
Source location	University of Washington Friday Harbor Laboratories, Friday Harbor, Washington, USA (48.5479° N, 123.0142° W)
Genome size (bp)	4,368,354
G + C content (%)	41.5
Mean coverage (×)	797
No. of raw reads (Illumina)	22,547,344
No. of raw reads (Nanopore)	515,036
No. of contigs	5 (Contigs 1–5)
N50 (bp)	1,415,317
No. of genes	4,024
No. of coding sequences (total)	3,903
No. of coding sequences (with protein)	3,735
No. of rRNAs	8, 9, 8 (5S, 16S, 23S)
No. of tRNAs	92
No. of ncRNAs	4
Locus positions of genes annotated to encode aerolysin-like toxins (e.g., aerolysin family beta-barrel pore-forming toxin)	195,744–197,188 In contig 1
	420,801–422,249 In contig 3
	1,428,404–1,429,882 In contig 3

Annotation of the genome using the NCBI Prokaryotic Genome Annotation Pipeline v6.9 (9) predicted 3,903 coding sequences, and 25 rRNA, 4 ncRNA, and 92 tRNA genes, including 3 identified to encode proteins with sequence similarity to aerolysin (e.g., aerolysin family beta-barrel pore-forming toxin) (Table 1); a well-known pore-forming toxin that can disrupt cellular membranes and is commonly associated with virulence (10–13). Given FHCF-3 is the causative agent of SSWD (14), these aerolysin-like toxins may play a role in the disease process.

**Fig 1:**
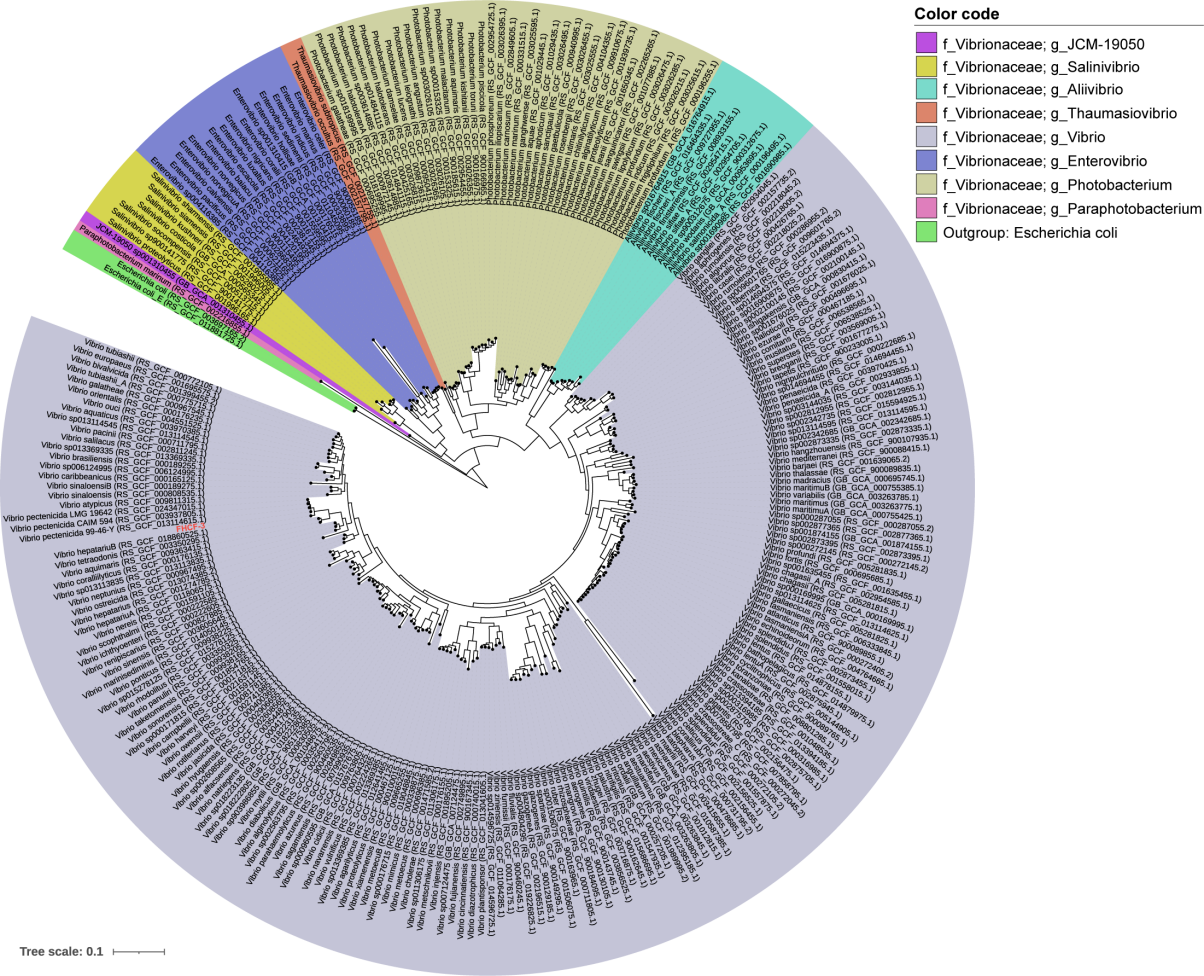
Phylogenomic tree of bacteria in the family Vibrionaceae. This phylogenomic tree illustrates the placement of strain FHCF-3 within the family Vibrionaceae, using genome data extracted from the Genome Taxonomy Database (GTDB, version 217) and NCBI Reference Sequence Database (as of 14 November 2024). The tree was constructed from genome sequences using GTDBtk v.2.3.2 (15) and rooted using *Escherichia coli* (GTDBtk accession numbers: RS_GCF_011881725.1 and RS_GCF_003697165.2) as an outgroup. Strain FHCF-3 is highlighted in red text.

To determine the evolutionary relationship of strain FHCF-3 to other bacteria, a whole-genome phylogeny was constructed using GTDBtk v.2.3.2 (15). Strain FHCF-3 clustered in a well-supported clade (bootstrap support, 99.8/100) comprising members of *Vibrio pectenicida* (Fig. 1).

Default parameters were used for all software.

## Data Availability

The genome sequence of *V. pectenicida* strain FHCF-3 was deposited in GenBank under accession number JBLZMR000000000. Raw Nanopore and Illumina sequencing reads were deposited in NCBI SRA database under accession numbers SRR32620450 and SRR32620451, respectively. Strain FHCF-3 is available upon request from the corresponding authors.

## References

[1] Hamilton SL, Saccomanno VR, Heady WN, Gehman AL, Lonhart SI, Beas-Luna R, Francis FT, Lee L, Rogers-Bennett L, Salomon AK, Gravem SA. 2021. Disease-driven mass mortality event leads to widespread extirpation and variable recovery potential of a marine predator across the eastern Pacific. Proc Biol Sci 288:20211195. doi:10.1098/rspb.2021.1195 34428964 PMC8385337

[2] Suttle CA, Chen F. 1992. Mechanisms and rates of decay of marine viruses in seawater. Appl Environ Microbiol 58:3721–3729. doi:10.1128/aem.58.11.3721-3729.1992 16348812 PMC183166

[3] Middelboe M, Chan AM, Bertelsen SK. 2010. Isolation and life cycle characterization of lytic viruses infecting heterotrophic bacteria and cyanobacteria, p 118–133. In Wilhelm SW, Weinbauer MG, Suttle CA (ed), Manual of aquatic viral ecology. ASLO.

[4] Zhong KX, Chan AM, Al-Qattan A, Li Y, Suttle CA. 2023. Complete genome sequence of Vibrio natriegens strain PWH3a. Microbiol Resour Announc 12:e0110822. doi:10.1128/mra.01108-22 36598262 PMC9872580

[5] Illumina. 2019. bcl2fastq: a proprietary Illumina software for the conversion of bcl files to basecalls. Available from: https://support.illumina.com/sequencing/sequencing_software/bcl2fastq-conversion-software.html

[6] Wick R. 2018. Porechop: adapter trimmer for Oxford Nanopore reads. GitHub. https://github.com/rrwick/Porechop.

[7] Wick RR, Judd LM, Gorrie CL, Holt KE. 2017. Unicycler: resolving bacterial genome assemblies from short and long sequencing reads. PLoS Comput Biol 13:e1005595. doi:10.1371/journal.pcbi.1005595 28594827 PMC5481147

[8] Parks DH, Imelfort M, Skennerton CT, Hugenholtz P, Tyson GW. 2015. CheckM: assessing the quality of microbial genomes recovered from isolates, single cells, and metagenomes. Genome Res 25:1043–1055. doi:10.1101/gr.186072.114 25977477 PMC4484387

[9] Li W, O’Neill KR, Haft DH, DiCuccio M, Chetvernin V, Badretdin A, Coulouris G, Chitsaz F, Derbyshire MK, Durkin AS, Gonzales NR, Gwadz M, Lanczycki CJ, Song JS, Thanki N, Wang J, Yamashita RA, Yang M, Zheng C, Marchler-Bauer A, Thibaud-Nissen F. 2021. RefSeq: expanding the Prokaryotic Genome Annotation Pipeline reach with protein family model curation. Nucleic Acids Res 49:D1020–D1028. doi:10.1093/nar/gkaa1105 33270901 PMC7779008

[10] Los FCO, Randis TM, Aroian RV, Ratner AJ. 2013. Role of pore-forming toxins in bacterial infectious diseases. Microbiol Mol Biol Rev 77:173–207. doi:10.1128/MMBR.00052-12 23699254 PMC3668673

[11] Parker MW, van der Goot FG, Buckley JT. 1996. Aerolysin — the ins and outs of a model channel-forming toxin. Mol Microbiol 19:205–212. doi:10.1046/j.1365-2958.1996.355887.x 8825766

[12] Popoff MR. 2014. Clostridial pore-forming toxins: powerful virulence factors. Anaerobe 30:220–238. doi:10.1016/j.anaerobe.2014.05.014 24952276

[13] Peraro MD, van der Goot FG. 2016. Pore-forming toxins: ancient, but never really out of fashion. Nat Rev Microbiol 14:77–92. doi:10.1038/nrmicro.2015.3 26639780

[14] Prentice MB, Crandall G, Chan AM, Davis KM, Hershberger P, Finke JF, Hodin J, McCracken A, Kellogg CTE, Carvalho R, Prentice C, Zhong KX, Harvell D, Suttle CA, Gehman ALM. 2025. Vibrio pectenicida strain FHCF-3 is a causative agent of sea star wasting disease. Nat Ecol Evol. doi:10.1038/s41559-025-02797-2 40760083

[15] Chaumeil PA, Mussig AJ, Hugenholtz P, Parks DH. 2019. GTDB-Tk: a toolkit to classify genomes with the Genome Taxonomy Database. Bioinformatics 36:1925–1927. doi:10.1093/bioinformatics/btz848 31730192 PMC7703759

